# Image classification by addition of spatial information based on histograms of orthogonal vectors

**DOI:** 10.1371/journal.pone.0198175

**Published:** 2018-06-08

**Authors:** Bushra Zafar, Rehan Ashraf, Nouman Ali, Mudassar Ahmed, Sohail Jabbar, Savvas A. Chatzichristofis

**Affiliations:** 1 Department of Computer Science, National Textile University, Faisalabad, Pakistan; 2 Department of Software Engineering, Mirpur University of Science & Technology, Mirpur, Azad-Kashmir, Pakistan; 3 Department of Information Science, Neapolis University, Paphos, Cyprus; Shenzhen University, CHINA

## Abstract

The Bag-of-Visual-Words (BoVW) model is widely used for image classification, object recognition and image retrieval problems. In BoVW model, the local features are quantized and 2-D image space is represented in the form of order-less histogram of visual words. The image classification performance suffers due to the order-less representation of image. This paper presents a novel image representation that incorporates the spatial information to the inverted index of BoVW model. The spatial information is added by calculating the global relative spatial orientation of visual words in a rotation invariant manner. For this, we computed the geometric relationship between triplets of identical visual words by calculating an orthogonal vector relative to each point in the triplets of identical visual words. The histogram of visual words is calculated on the basis of the magnitude of these orthogonal vectors. This calculation provides the unique information regarding the relative position of visual words when they are collinear. The proposed image representation is evaluated by using four standard image benchmarks. The experimental results and quantitative comparisons demonstrate that the proposed image representation outperforms the existing state-of-the-art in terms of classification accuracy.

## 1 Introduction

One of the most challenging task in computer and robotics vision is to classify images into semantic categories [1]. Image classification refers to labelling the images with one of the pre-defined semantic category [2]. The challenges that make image classification a difficult task are the change in viewpoint, illumination, partial occlusion, clutter, inter and intra-class visual diversity. To deal with image classification, Bag-of-visual-words (BoVW) model attracted attention in the research community and proved to be a leading strategy [3]. It is widely used in literature to deal with problems such as image classification, retrieval, automatic image annotation and object recognition [4–14].

In the standard BoVW model, local features are extracted from a set of training images and quantized into visual words. The images are represented by histograms of visual words. This representation is orderless as histogram is the count of the number of times a word occurs in the image. It does not contain details about the location of visual words in 2-D image space [15, 16].

Various approaches are proposed in the literature to incorporate the spatial information to BoVW model [15, 17–20]. Some of these add spatial information by using the spatial context prior to the construction of visual vocabulary [21, 22]. Broadly they can be classified into two groups [3, 23, 24]. The first group encompasses methods that divides an image into sub-regions of different shapes and the information about visual words are computed from each of the selected region. Lazebnik *et al*. [15] proposed a notable contribution in this domain and proposed the Spatial Pyramid Matching (SPM). It divides the image space into rectangular sub-regions in a hierarchically decreasing order. To attain improved performance, visual words statistics are then aggregated from each rectangular region at each level on the basis of a weighed scheme [15]. However, SPM captures information only about the approximate geometric correspondence of visual words and is not invariant to global geometric transformations [25]. To achieve better performance, the authors used different approaches to incorporate additional spatial information into the SPM. Zhang *et al*. [26] proposed log-polar tiling, where the image space is partitioned into regions of different scales and orientations. Visual words statistics are compiled from each sector of the tiling to create the histogram. In another work [27], Zhang *et al*. proposed different heuristic approaches by employing three frequency histograms i.e. shapes, pairs and binned log-polar features representation. To attain the photometric image aspects, Yang *et al*. [28] captured the spatial information that is based on the co-occurrence information to ascertain the geometric and photometric image aspects. Word Spatial Arrangement (WSA) [29] is another method that infuses the relative spatial position of visual words by defining each point as origin and partitions the image space into quadrants. Koph *et al*. [30] enhanced the classification performance of BoVW model by incorporating color pyramids in place of spatial pyramids. Instead of dividing the image into spatial tilings, it is divided on the basis of color information of pixels. BoVW with SPM is sensitive to the changes in viewpoint and rotations [1]. Zhao *et al*. [31] proposed a concentric circle structured multi-scale BoVW using multiple features i.e. color moments, SIFT and Local Binary Patterns (LBPs).

The second group comprises of methods that encode relationships [18, 25] or co-occurrence of visual words [32]. The modeling of geometric spatial relationships between visual words received relatively little attention as it is computationally expensive [25, 33]. To accelerate the computation, this category uses techniques to reduce the size of visual vocabulary or employs some feature selection techniques. Savarese *et al*. [18] calculated correlogram to represent relationships among visual words. As correlogram is a function of distance, the choice of distance measures affect the outcome and makes this representation vulnerable to scale changes. Khan *et al*. [25] made a notable contribution in this domain and incorporated global spatial information in BoVW model by considering the global geometric relationships among the Pairs of Identical Words (PIWs). A normalized histogram is created that is based on angles between these identical visual words termed as PIWAH (Pairs of Identical Visual Words Angle Histogram). The PIWAH representation is invariant to geometrical transformations i.e. scaling and translation but is sensitive to rotation variance [23, 25]. Anwar *et al*. [23] extended this work to acquire rotation invariant geometric properties, by considering the orientation of segments formed by Triplets of Identical Visual Words (TIWs). The histogram representation so created is termed as TIWAH (Triplets of Identical Visual Words Angle Histogram).

**Fig 1:**
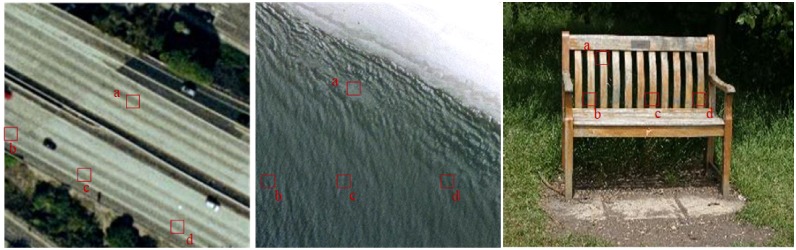
Representation of distribution of collinear points in images.

Though the approach of Anwar *et al*. [23] using angles between identical visual word triplets achieves rotation invariance but loses the information regarding the relative position of points when they are collinear as can be seen in Fig 1. This article presents a novel way to model global relative spatial orientation of visual words in a rotation invariant manner. For this we computed the geometric relationship between triplets of identical visual words by calculating an orthogonal vector relative to each point in the triplets of identical visual words and calculating the histogram on the basis of the magnitude of these orthogonal vectors. The major contributions of this paper are i) adding the discriminative global spatial information to the BoVW model ii) being robust to geometric transformations such as rotation. Experimental outcomes on standard benchmarks demonstrate remarkable gain in the classification accuracy over the state-of-the-art methods.

The rest of the article is organized as follows: the proceeding section is about the literature review. Section 3 provides an overview of the BoVW model and presents our proposed approach to incorporate the global spatial information to the inverted index of BoVW model. Section 4 provides a discussion about results on four benchmark datasets, and comparison with the other state-of-the-art. The last section concludes the article and points towards the future directions of research.

## 2 Related work

A major limitations of BoVW model is that it ignores spatial information [8, 25]. Despite of this fact, BoVW exhibits high discriminative power and shown excellent results in image classification [14, 34, 35]. Other challenges faced by the BoVW representation are the lack of semantic meaning and performance evaluation of BoVW-based systems, which are open areas of research [36–39]. Numerous research studies demonstrated that the performance can be improved by incorporating the missing spatial information [15, 40, 41]. The most notable work in the context of spatial information is of Lazebnik *et al*. [15] (who proposed SPM). In SPM, an image is divided into rectangular subregions and visual word statistics are aggregated from each region. The final histogram is the concatenation of histograms extracted from each region. To reduce the dimensions of feature vector extracted from SPM, [40, 41] proposed to incorporate the spatial context at a lower level. Koniusz *et al*. [40] put forward Spatial Coordinate Coding (SCC), to encode the spatial and angular information at descriptor level. Krapac *et al*. [42] proposed a framework to derive a compact feature representation, that encodes the spatial layout of visual words using a Gaussian Mixture Model (GMM). A similar approach was proposed by SáNchez *et al*. [41] to include spatial information in image signatures on the basis of average statistics. A significant benefit of these approaches when compared with SPM is they do not incur an increase in the dimensions of image representation. Object Bank (OB) is a high-level image representation that encodes the spatial and semantic information [43]. However OB approach suffers from drawback of high-dimensionality and various approaches have been proposed in literature to reduce the dimensions and enhance the performance of OB [43, 44]. To boost the performance of OB representation Zang *et al*. [44] proposed a threshold value filter method. They used Matthew effect normalization method to simplify OB representation and constructed more compact descriptors. They showed improved performance on three real-world datasets, with substantial dimensionality reduction of image descriptors.

To prove the effectiveness of proposed research, besides methods concurrent to our approach [18, 23–25], we have selected some recent state-of-the-art focussed on different approaches as feature fusion [1, 2], intermediate feature representation [45], the use of Convolutional Neural Networks (CNN) and deep learning techniques [46, 47] to improve the classification performance. In [1], Zou *et al*. proposed local-global-fusion strategy (LGF), to create a fusion of local and global image features. For this they first extracted local features by using BoVW and SPM, in order to extract global features they employed multi-scale CLBP (MS-CLBP). For feature representation they employed Kernel Collaborative Representation-based Classification (KCRC). After the representation residuals are obtained from the two types of features, the label is assigned based on the sum of the weighed residuals.

In another recent work, Bian *et al*. [2] proposed fusion of local and global descriptors to enhance the classification performance. They enriched the feature representations by combining both global structures and local fine details of image scene. To extract global rotation-invariant features they employed global saliency based multiscale, multiresolution and multistructure LBP, and local Codebookless Model (CLM) is used to represent local discriminative features. They reported improved performance to their complementary as well as more competitive state-of-the-art deep learning methods. Mekhalfi *et al*. proposed a novel scheme to compactly represent images using a compressive sensing and multi-feature framework. Their method achieved substantial performance gains results to the state-of-the-art methods on land-use image dataset.

Recent works show the effectiveness of deep learning methods on scene classification [46, 48]. A major limitation of CNN based architectures is the complicated pre-training process for fine-tuning parameters [2]. Zhang *et al*. [48] proposed a Gradient Boosting Random Convolutional Neural Network (GBRCN) framework for image classification. They effectively combined many deep neural networks to cerate a deep ensemble network for the first time. They performed experiments on two challenging high-resolution datasets and provided accurate results than the state-of-the-art methods. To accelerate learning of deep CNNs, Scott *et al*. [46] proposed to use Transfer Learning (TL) in combination with fine-tuning and augmentation. They evaluated the effectiveness of proposed approach on UC Merced dataset to achieved significantly higher accuracies than the most outstanding methods. It is worth mentioning here, that for these datasets CNN based approaches [47] are not an optimal choice, as they require huge amounts of data (in millions) and time for training [24]. The BoVW model is a plug-n-play method which can be adopted without any prior initialization or training [4].

## 3 Proposed methodology

This section is about an overview of BoVW model and introduce its basic notations, then we will discuss the proposed Orthogonal Vector Histograms (OVH) representation and the implementation details.

### BoVW model

BoVW is analogous to the Bag-of-Words (BoW) used in textual retrieval systems [49]. The BoW representation of a document is a normalized histogram that counts of the occurrences of a word in a document. The resultant BoW representation is also termed as ‘bag’, as it keeps only the count and does not retain the order of words in the document. Histogram intersection is used to determine similarity. If the images are different, the result of their intersection is small. In English language, there is a vocabulary of words but for images, we need to create our own vocabulary. The words in images are little picture elements, just as document words, the features represent the local areas of the image. In BoVW, an image *Im* is depicted as a set of image descriptors as Eq (1)

$$\begin{array}{c} Im=\{d_{1},d_{2},d_{3},....,d_{I}\} \end{array}$$ (1)

where *d*_*i*_ is the color, shape, and *I* denotes total image descriptors.

As a result, numerous local descriptors are created from all the patches of each image for a given dataset. To reduce the dimensions of resultant feature vectors, an unsupervised clustering technique *k*-means [49] is applied on the extracted descriptors to find cluster centers that constitute the visual vocabulary

$$\begin{array}{c} v=\{w_{1},w_{2},w_{3},....,w_{K}\} \end{array}$$ (2)

where *K* is the predefined number of clusters or visual words an *v* is the constructed vocabulary of code book.

So mapping of each descriptor to the nearest visual word is done according to the Eq (3)

$$\begin{array}{c} w\left(d_{j}\right)=arg\underset{w\in v}{min}Dist\left(w,d_{j}\right) \end{array}$$ (3)

Here, w(*d*_*j*_) depicts the visual word assigned to *j*^*th*^ descriptor and *Dist* (w,*d*_*j*_) signifies the distance between the descriptor *d*_*j*_ and visual word *w*.

Clustering is required to reduce the high dimensional feature space to obtain a more compact feature representation. Each image is hence represented by a collection of descriptors, with each descriptor mapped to one visual word. In the conventional BoVW method [49], the histogram is the final representation of the image which gives the distribution of visual words. It does not have any order. The count of bins in histogram equals the number of visual words in the dictionary (i.e. *K*). If each bin represents a visual word *w*_*i*_ in *voc*

$$\begin{array}{c} bin_{i}=card\left(D_{i}\right)\;\;where\;\;D_{i}=\left\{d_{j},j\in 1,....,n|w\left(d_{j}\right)=w_{i}\right\} \end{array}$$ (4)

*D*_*i*_ is the set of all the descriptors that correspond to a particular visual word *w*_*i*_ in an image. *Card*(*D*_*i*_) is the cardinality which gives count of the elements of set *D*_*i*_. This is repeated for every word in image to obtain the final representation. The histogram hence created does not retain the spatial information of the interest points.

### Orthogonal Vectors Histogram (OVH)

**Fig 2:**
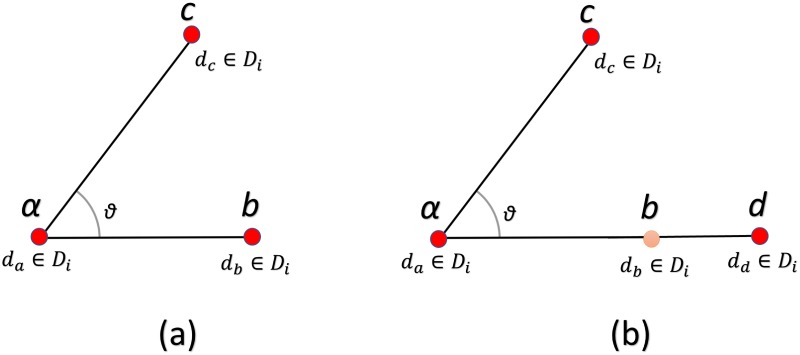
(a) Depicts the angle for descriptor *d*_*a*_ relative to *d*_*b*_ (b) the angle for descriptor *d*_*a*_ relative to descriptor *d*_*d*_.

BoVW model assigns identical image patches to the same visual word to create the histogram representation of images. Khan *et al*. [25] proposed to model global relationship between visual words by using PIWs to describe images where a given pair corresponds to two identical words. The angles made by the position of PIWs are computed with respect to x-axis to create the PIWAH representation. Since the angles between PIWs are computed with respect to x-axis, PIWAH is not invariant to rotation [23, 25]. To acquire rotation invariance Anwar *et al*. [23] proposed to compute angles between TIWs. The angles hence computed between TIWs are used to create the TIWAH representation. Though the approach of Anwar *et al*. [23] using angles between identical visual word triplets achieves rotation invariance but loses fine information regarding the relative position of points when they are collinear. As we can see in Fig 2 the position of *b* and *d* is different relative to *a*, but the angle at point *a* relative to *b* and *d* is same.

**Fig 3:**
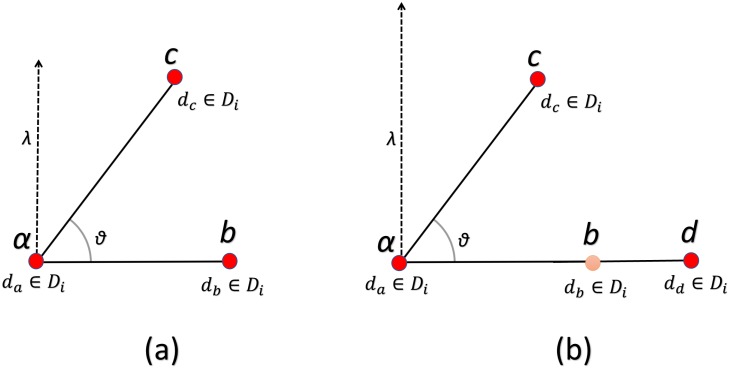
(a) The orthogonal vector for descriptor *d*_*a*_ relative to *d*_*b*_ (b) the orthogonal vector for descriptor *d*_*a*_ relative to descriptor *d*_*d*_.

It is obvious from Fig 2 that the angle at point *a* relative to *b* and *d* is same, despite the fact that their relative positions are different with respect to point *a*. This results in loss of spatial information and decreases the discriminative power of the model. We proposed a novel approach to incorporate global spatial information by calculating an orthogonal vector relative to each point in the triplets of identical visual words as shown in (Fig 3) and calculating the histogram on the basis of the magnitude of these orthogonal vectors.

If the points are collinear their relative angle will be the same but the magnitude of their orthogonal vector will be different. Our approach adds the spatial information to the BoVW model and hence increases the discriminative power of the model.

**Fig 4:**
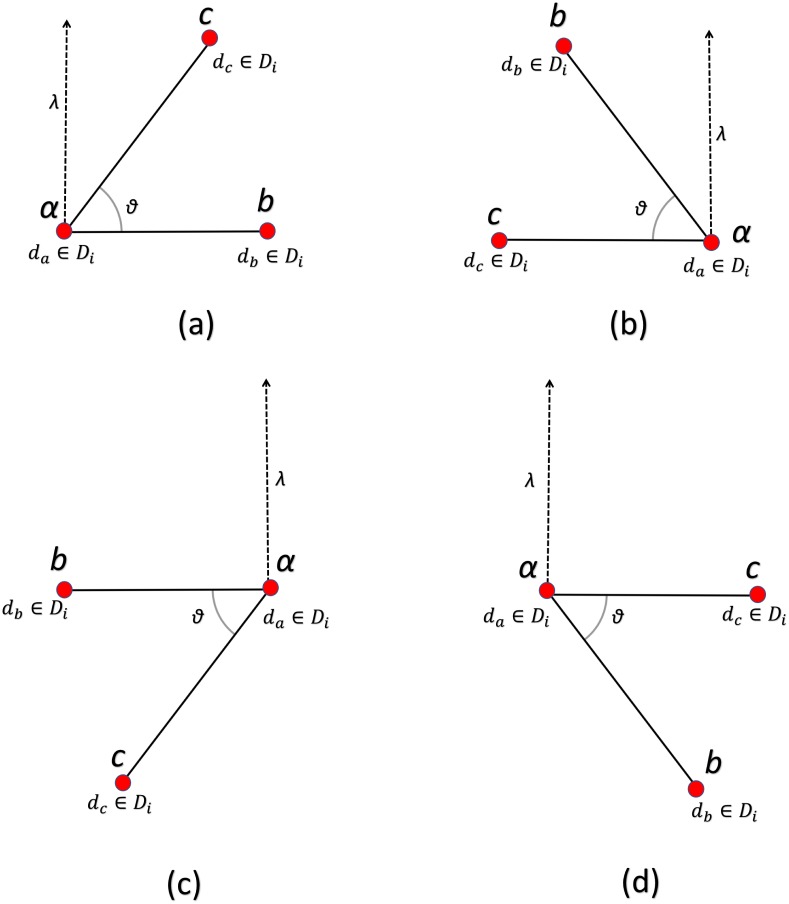
Figure depicts the rotation-invariance achieved between point triplets using the magnitude of orthogonal vectors.

If the image is rotated by any degree the orthogonal vector between point triplets will remain the same thereby achieving rotation invariance as can be seen in Fig 4.

Hence we define the set of all triplets (TW) of identical visual words related to a visual word *w*_*i*_ as:

$$\begin{array}{c} TW_{i}=\left\{\left(a,b,c\right)|\left(d_{a},d_{b},d_{c}\right)\in D_{i}^{3},d_{a}\neq d_{b}\neq d_{c}\right\} \end{array}$$ (5)

where *a*(*a*_1_, *a*_2_), *b*(*b*_1_, *b*_2_) and *c*(*c*_1_, *c*_2_) signify the spatial positions of the descriptors *d*_*a*_, *d*_*b*_ and *d*_*c*_ respectively. The position of a descriptor is determined by coordinates of the top left pixel of the relevant patch. As *i*th bin of histogram represents *d*_*i*_, its value gives the frequency of occurrence of word *w*_*i*_. The cardinality of *TW*_*i*_ is ${}^{b_{i}}C_{3}$ i.e. the number of possible combinations between distinct vector triplets among *b*_*i*_ elements.

The position vectors of *b* and *c* with respect to *a* are given by:

$$\begin{array}{c} r_{ab}=\left(b_{1}-a_{1},b_{2}-a_{2}\right)\\ r_{ac}=\left(c_{1}-a_{1},c_{2}-a_{2}\right) \end{array}$$

Let $P_{a}^{bc}$ denotes the vector at *a* orthogonal to **r**_*ab*_ and **r**_*ac*_, then

$$\begin{array}{cc} P_{a}^{bc} & =r_{ab}\times r_{ac}\\ & =|\begin{array}{cc} \hat{i} & \hat{j}\\ b_{1}-a_{1} & b_{2}-a_{2}\\ c_{1}-a_{1} & c_{2}-a_{2} \end{array}|\\ & =(\left(b_{1}-a_{1}\right)\left(c_{2}-a_{2}\right),\left(b_{2}-a_{2}\right)\left(a_{1}-c_{1}\right)) \end{array}$$

The magnitude of $P_{a}^{bc}$ is calculated as

$$\begin{array}{c} |P_{a}^{bc}|=\sqrt{{\left[\left(b_{1}-a_{1}\right)\left(c_{2}-a_{2}\right)\right]}^{2}+{\left[\left(b_{2}-a_{2}\right)\left(a_{1}-c_{1}\right)\right]}^{2}} \end{array}$$ (6)

The magnitude of these orthogonal vectors are scaled in the range of 0-1. The orthogonal vector histogram *OVH*_*i*_ provides the spatial distribution for a particular visual word *w*_*i*_. To obtain a global representation, we combined *OVH*_*i*_ obtained from all the visual words in an image. For this we used a bin replacement technique, to transform the BoVW for OVH representation. This is done by replacing each bin of BoVW frequency histogram with the *OVH*_*i*_ histogram corresponding to *w*_*i*_. To incorporate the spatial information by keeping the frequency information intact, we normalized the sum of all bins of *OVH*_*i*_ to the bin-size *b*_*i*_ of the respective bin of BoVW histogram that is going to be replaced. The global representation of an image, denoted by OVH is hence formulated as

$$\begin{array}{c} OVH=(\alpha_{1}OVH_{1},\alpha_{2}OVH_{2},.....,\alpha_{K}OVH_{K}) \end{array}$$ (7)

where $\alpha_{1}=\frac{b_{i}}{||OVH_{i}\left|\right|}$ and is termed as the coefficient of normalization. For a visual vocabulary of size K, if the number of histogram bins is H, then the size of OVH is *K* × *H*.

### Implementation details

**Fig 5:**
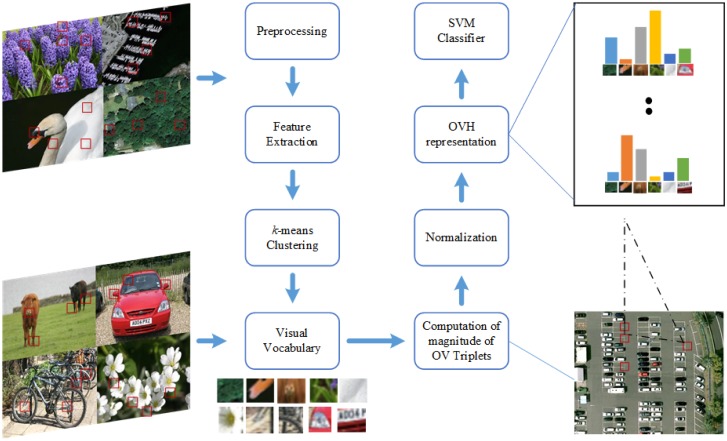
Block diagram of proposed research based on OVH.

The block diagram of the proposed methodology is shown in Fig 5. For all datasets, we followed the same sequence of steps to create histogram representations. To reduce the computational complexity, as a pre-processing step, the large images from datasets are resized to a standard size of 480 × 480 pixels. For feature extraction, all the images are converted to gray scale and dense SIFT with step size of 8 is used for feature extraction. Then *k-means* clustering is applied on these descriptors to generate visual vocabulary. Due to unsupervised nature of *k-means*, the experiments are repeated 10 trials with random selection of training and test images and mean values are reported in tables and graphs.

**Fig 6:**
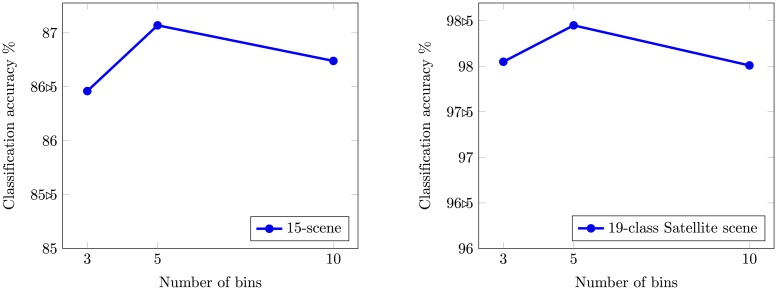
Mean average classification accuracy as a function of bin size.

The size of visual vocabulary is an important parameter affecting the performance of system. Increasing the size of visual vocabulary increases the performance and a larger vocabulary size tends to overfit [50]. Experiments are conducted with vocabulary of different sizes in-order to determine the best performance obtained from the proposed image representation. To speed up computation, we set a threshold and a random selection is used to limit the number of words of the same type used for the creating triplet combinations. We used 5-bin OVH representation for the results presented in section 4. Fig 6 presents the empirical justification for the choice of optimal bins for histogram representation on two datasets used in our experiments. We performed experiments for proposed research and TIWAH [23] following the same experimental parameters.

For classification we have used Support Vector Machines (SVM) that belongs to supervised learning methods [51]. Given positive and negative training images, the objective is to classify a test image whether it contains the object class or not. SVM uses the kernel method to calculate the dot product in the high dimensional feature space and acquires the ability to generate non-linear decision boundaries. The kernel method makes it possible to use data with no obvious fixed dimensions. The histograms constructed by computing the magnitude between orthogonal vectors are normalized and SVM Hellinger Kernel [52] is applied to the normalized histograms. The SVM Hellinger kernel is selected because of its low computational cost and instead of computing the kernel values it explicitly computes the features map and the classifier remains linear [6]. To determine the optimal value for the regularization parameter *C*, 10-fold cross validation is applied on the training dataset. The one-against-one [53] approach is applied and for *k* number of classes, *k*.(*k*-1)/2 classifiers are constructed to train the data using two classes.

## 4 Experiments and results

This section provides details about the experiments that are conducted for the evaluation of proposed image representations. To evaluate the effectiveness of proposed research, experiments are conducted on standard datasets that are used extensively in the literature.

### 15-scene image dataset

**Fig 7:**
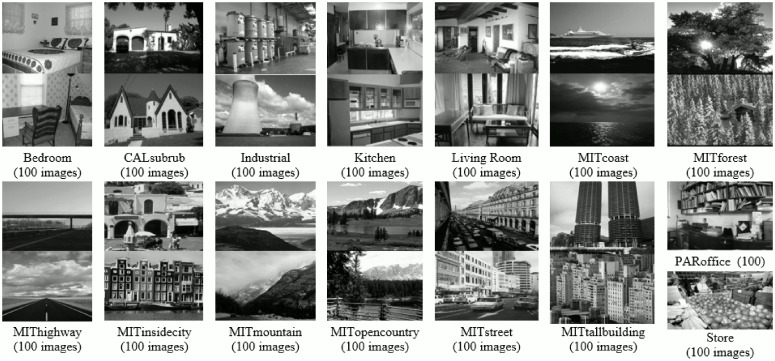
Example images with class label and total images per class, for each category of 15-scene image dataset [15].

The first dataset used in our experiments comprises of 15-scene categories. Initial 8 categories are contributed by Oliva and Torralba [54], 5 classes are collected by Li and perona [34] and the rest are introduced by Lazebnik [15]. Images are collected from different sources primarily from personal photographs, the Internet and COREL collections. The total number of images in his dataset are 4485 and average image size is 300 × 250 pixels, with 210-410 images per category. It is a challenging dataset, as it comprises of a wide range of indoor and outdoor categories as can be seen in Fig 7.

For this dataset, we followed the same experimental procedure as mentioned in [15, 25]. To ensure a fair comparison, the testing and training samples are chosen in accordance with the state-of-the-art methods. The training set comprises of 100 randomly selected images and the rest of the images are used for testing as the same number is elected by the papers that are used for comparison.

**Fig 8:**
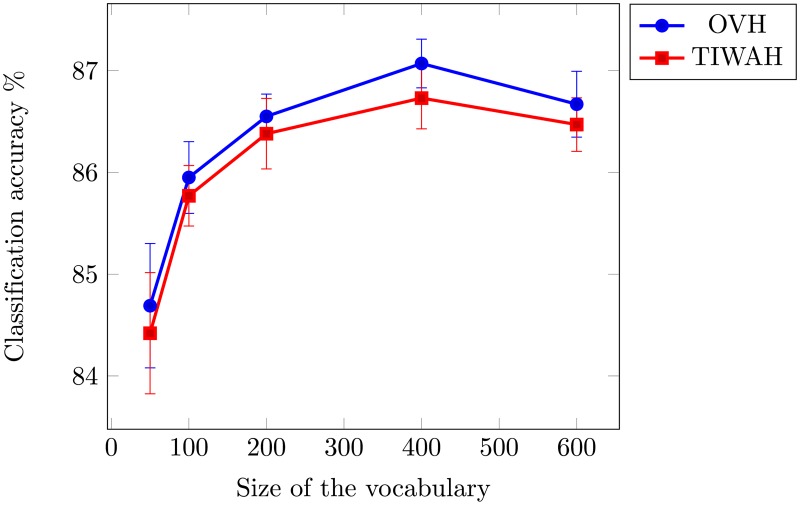
Mean average accuracy as a function of vocabulary size using 15-scene image dataset.

We performed experiments with different sizes of visual vocabulary to obtain the optimal size for accurate feature representations. The mean and standard deviation over 10 individual runs are shown in Table 1. For PIWAH [25] the best mean average accuracy was reported for a vocabulary size of 200. For our experiments, we obtained best performance for OVH and TIWAH representation for a vocabulary size of 400. Fig 8 provides a graphical comparison over vocabulary of different sizes (with 95% Confidence Interval (*CI*)).

**Table 1:** Classification accuracy comparison with PIWAH, TIWAH and proposed research.

Voc. Size	PIWAH		TIWAH		OVH	
	*μ*	*σ*	*μ*	*σ*	*μ*	*σ*
100	74.6%	0.6	85.77%	0.42	85.95%	0.49
200	76.0%	0.6	86.38%	0.48	86.55%	0.31
400	75.9%	0.6	86.73%	0.42	87.07%	0.33

Experimental results and comparisons show the robustness of the proposed approach using 15-scene image dataset. In Table 2 we provide a comparison of the proposed OVH representation with the state-of-the-art based on spatial context. The most notable contribution in the context of spatial information is of Lazebnik *et al*. [15]. Savarese and Liu [17, 18] are the poineers of pairwise spatial histograms, in which all the possible combinations of visual words are considered leading to *N*(*N* + 1)/2 histograms (where N denotes the number of visual words). In [18], only distance divisions are considered, whereas [17] combines both angle and distance information. Our work relates most closely to PIWAH [25] and TIWAH [23] as we have modeled global relative geometric relationships between identical visual words. In PIWAH, only relationships between identical visual words are considered resulting in *N* spatial histograms. Anwar *et al*. [23] proposed to compute angle between triplets of identical visual words to acquire rotation invariance. The experimental results show that our proposed method outperforms the state of the art methods in both accuracy and dimensions.

**Table 2:** Classification accuracy comparison of the proposed research with the state-of-the-art methods.

Algorithms	Feature Dimensionality	Accuracy
PIWAH [25]	1800	76%
SPM [15]	8400	81.4%
Zang *et al*. [44]	3717	81.5%
PIWAH+ [25]	5000	82.5%
SPS_ad_+ [24]	13200	83.7%
EMFS [55]	X	85.7%
LGF [1]	X	85.8%
TIWAH	3600	86.73%
OVH	2000	**87.07%**

Here, it is important to note that for PIWAH [25] best performance i.e. 76% is obtained for visual vocabulary of size 200, and the dimensions of the resultant feature vector are 1800. For OVH we obtain the optimal performance on *voc* size of 400 i.e. 87.07% resulting in a 2000 dimensional feature vector. If performance of OVH is compared with PIWAH for *voc* size of 200, the dimensions of OVH are 1000 and accuracy is 86.55% (Table 1) which is still significantly higher than the PIWAH representation. Khan *et al*. [25] incorporate the absolute spatial information in PIWAH+, by combining the SPM and PIWAH and obtain a performance gain of 6.5% with a 5000 dimensional feature vector. SPS_ad_+ [24] enhanced the PIWAH representation by combining orientation, distance and SPM representation and obtained 83.7% on the tradeoff of dimensionality, which increased upto 13200. SPS_ad_^1800^+ reduced the dimensions of SPS_ad_+ to a 1800 dimensional feature vector, followed by a subsequent reduction in accuracy that drops by 0.7%.

Besides methods concurrent to our approach, we have also provided comparison with some of the recent works focused to enhance classification accuracy. In [55], Song *et al*. adopted a different approach to incorporate spatial context, i.e. by combining the semantic and the spatial information to create the Extended Mutli-feature Spatial Context (EMFS) and achieved 85.7% performance accuracy. Zou *et al*. [1] created a fusion of local (extracted by combining BoVW with SPM) and global (extracted using multi-scale CLBP) features and reported an accuracy of 85.8%. Another recent work [44] that combines the semantic and spatial context, reducing the dimensions of Object Bank (OB) to 1/12 obtains an accuracy of 81.5% with 3717 dimensions. OVH clearly outperforms the methods concurrent to our approach in both accuracy and dimensions.

**Fig 9:**
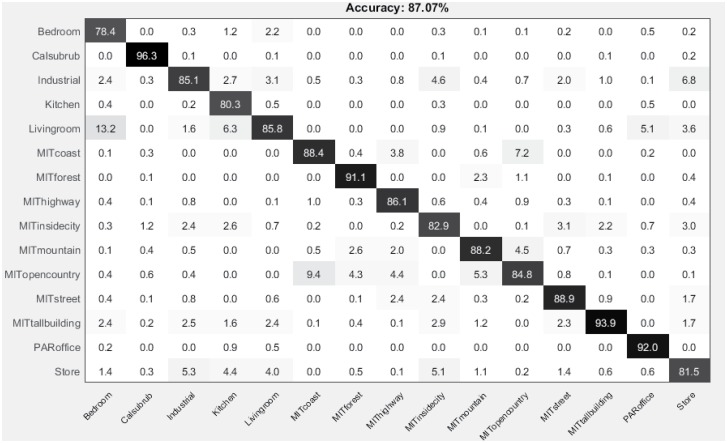
Confusion matrix for the 15-scene image dataset.

The confusion matrix for 15-scene dataset is shown in Fig 9. The diagonal values show the precision normalized percentages of each class.

The above comparisons clearly demonstrate that our approach outperforms the state-of-the-art spatial methods, with relative global spatial information only and no additional dimension reduction steps required.

### MSRC-v2 image dataset

**Fig 10:**
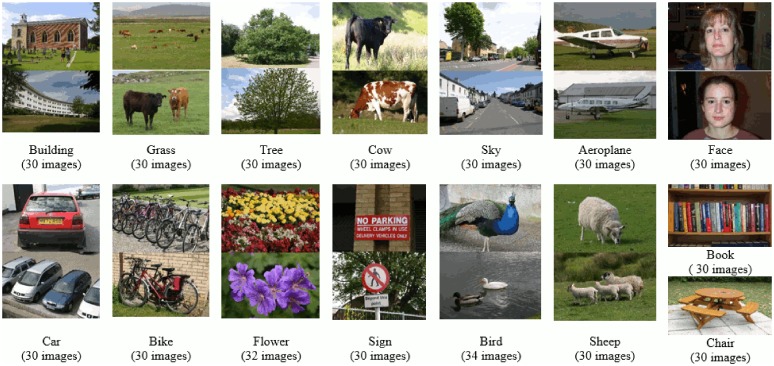
Example images with class label and total images per class, for each category of MSRC-v2 image dataset [18].

The second dataset used in our experiments consists of 591 images classified into 23 categories. Different subsets of these categories have been used in literature to evaluate a classification problem. For MSRC-v2 we have used a 15 category problem as in [17, 18, 24]. The training and test sets are chosen in accordance with these works to ensure fair comparison. It is a challenging dataset as the objects exhibit intra-class variation in shape and size, in addition to partial occlusion [17]. Example images from this dataset are shown in Fig 10.

**Fig 11:**
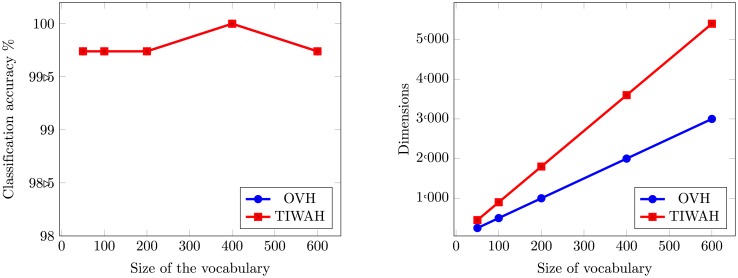
Performance comparison between TIWAH and OVH for MSRC-v2 image dataset.

To obtain the optimal size for feature representation, experiments are conducted with different sizes of visual vocabulary based on proposed OVH and TIWAH [23]. For PIWAH [25] the best mean average accuracy was reported for a vocabulary size of 400. The dimensions of resultant feature vector for PIWAH are 3600. For our experiments, we also obtained the best performance for OVH and TIWAH representation, for a vocabulary size of 400 as can be seen in Fig 11. The dimensions of TIWAH feature vector are 3600 and for OVH 2000 respectively.

The first part of the Fig 11 demonstrates the classification accuracy comparison between TIWAH and OVH, and the second part shows the dimensions of the resultant feature vector obtained from both representations. Though for MSRC-v2 dataset, the classification accuracy performance obtained from both methods is parallel, the dimensions of TIWAH obtained for the best performance are 1.8 times more as compared to OVH.

Table 3 provides a comparison of OVH to the methods that relate closely to our approach. Here, we can see that our method outperforms the related methods in terms of accuracy and dimensions. Savarese *et al*. [18] and Liu *et al*. [17] are the most notable contributions to model spatial relationships between visual words. In order to build spatial histograms they rely on new features comprising of pairs (or higher number) of words having a specific relative position. The approach of Savarese *et al*. results in 81.1% accuracy, and Liu *et al*. achieved 83.1% accuracy. Our method provides the best classification results for this dataset. Besides this, the proposed approach holds different other advantages compared to existing methods. Liu *et al*. [17] used integrated feature selection and spatial feature extraction technique to boost the performance. However, as spatial information extraction is performed as a part of learning step, the modification in the training set would lead to feature re-computation thus hence making it difficult to generalize. Unlike Savarese *et al*. [18], OVH does not require a 2^nd^-order feature quantization step.

**Table 3:** Classification accuracy comparison of the proposed research with the state-of-the-art methods.

Algorithms	Dimensions	Accuracy
Saverse *et al*. [18]	X	81.1%
PIWAH [25]	3600	82.0%
Liu *et al*. [17]	1200	83.1%
SPS_ad_ [24]	18000	83.5%
TIWAH	3600	100%
OVH	2000	**100%**

The soft pairwise similarity angle distance histogram (SPS_ad_) [24] encodes spatial information of pairwise similar patches into the BoVW representation. SPS_ad_ results in 83.5% accuracy with 18000 dimensional feature vector. Compared to SPS_ad_ our proposed representation provides 16.5% higher accuracy, with a low dimensional feature vector. The performance of TIWAH in this method is parallel to our method but its dimensions are almost 1.8 times more than OVH. Our proposed method clearly outperforms the state-of-the-art concurrent methods, by modeling global geometric relationship between visual words.

**Fig 12:**
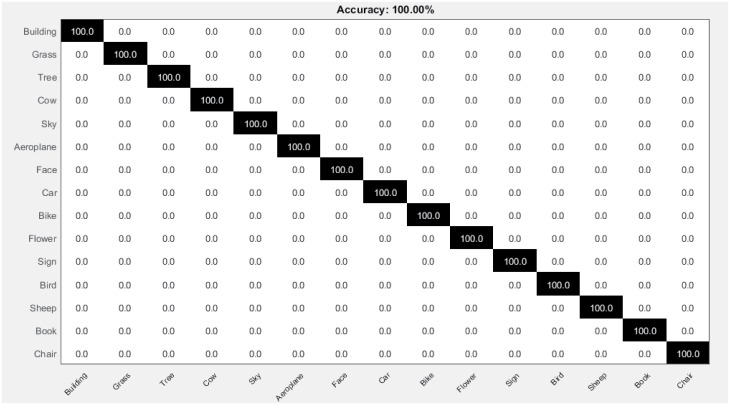
Confusion matrix for the MSRC-v2 image dataset.

The confusion matrix calculated from 10 runs of proposed OVH for its highest performance on 400 *voc* size is shown in Fig 12. It shows the robustness of proposed approach, that significantly enhances the performance by accurately classifying all images into their respective categories.

### UC Merced land-use (UCM) image dataset

**Fig 13:**
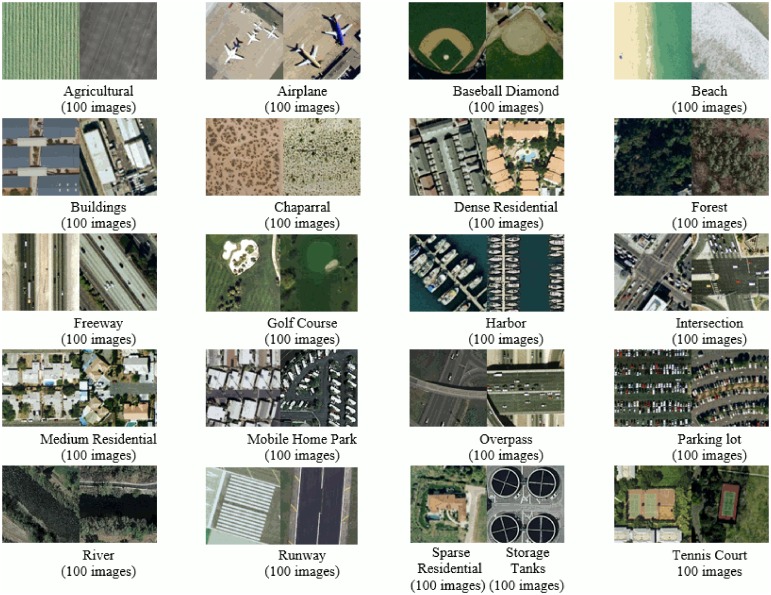
Example images with class label and total images per class, for each category of UCM image dataset [56].

The third dataset used in our experiments is created by Yang and Newsam [56] comprising of images downloaded from the United States Geological Survey (USGS) National map. It comprises of 21 land-use classes as shown in Fig 13. Each class contains 100 images of size 256 × 256 pixels. This benchmark dataset has a large geographical scale. Following the experimental setup in [1, 2, 56] we randomly selected 80 images from each class as training and the remaining for testing.

Experiments conducted on 15-scene and MSRC-v2 datasets clearly demonstrate that our method outperforms methods that do the same (incorporate spatial context) to our approach, based on spatial information. Here, to prove the effectiveness of our approach, comparison is performed with the state-of-the-art ranging from feature fusion [1, 2], intermediate feature representation [45], the application of CNN and deep learning techniques [46, 47].

**Fig 14:**
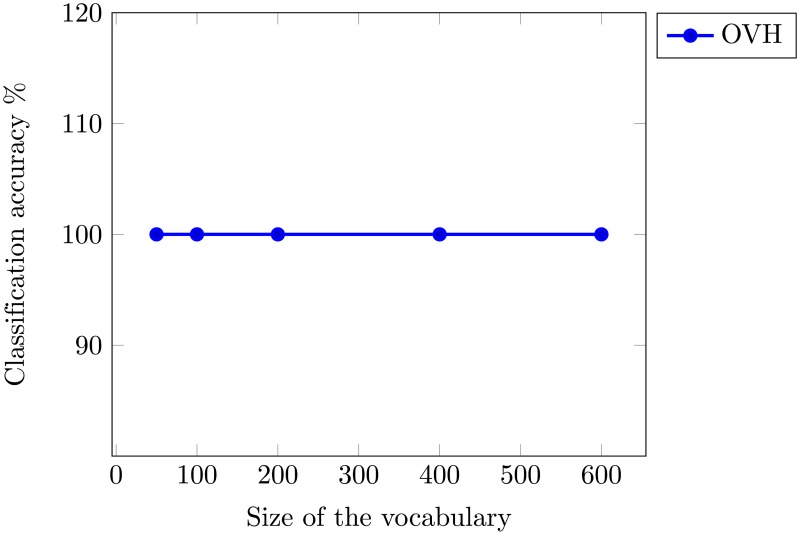
Mean average classification accuracy as a function of vocabulary size using UCM image dataset.

To obtain the optimal performance, the accuracy of OVH for different vocabulary sizes is shown in Fig 14. Even at vocabulary size of 50 our proposed method shows excellent performance with dimensions as low as 250. In-order to provide fair comparison with state-of-the-art we have used the same training and test ratio as in related works. It will be interesting to conduct experiments with different training and test ratios and analyze the performance in those scenarios.

As UCM is a widely used dataset [1, 45, 57], a few noteworthy recent results are reported in Table 4. In CCM-BOVW [31], the spatial information is incorporated by using a concentric-circle based approach, in addition to multiresolution images, they used multiple features i.e. SIFT, color moments and LBP to enhance feature representation. Their approach appear good only for the classes that are sensitive to orientations, as airplane, baseball diamond, golf course and storage tanks. Whereas, CCM-BOVW did not have a significant impact on categories, that have simple pattern and do not suffer from orientations as forest, river, agricultural and chapparal. Our method archives 100% accuracy for this dataset by incorporating the relative spatial information in a rotation-invariant manner.

**Table 4:** Classification accuracy comparison of the proposed research with the state-of-the-art methods.

Algorithms	Accuracy
SPM [15]	82.3% ± 1.48% [58]
CCM-BOVW [31]	86.64% ± 0.81%
MS-CLBP_1_ [59]	90.6% ± 1.4%
SOS [45]	94.33%
LGF [1]	95.48%
salM^3^LBP-CLM [2]	95.75% ± 0.80%
LGFBOVW [58]	96.88% ± 1.32%
ResNet50 [46]	98.5%
Evolved Sugeno [47]	99.33%
OVH	**100%**

LGF [1], salM^3^LBP-CLM [2] and LGFBOVW [58] create a fusion of local and global features for high spatial resolution (HSR) remote sensing imagery. OVH outperforms the above methods by 4.52%, 4.25% and 3.12% classification accuracy respectively. Besides this, LGF [1] also incorporates the spatial information by including SPM in implementation. Though discriminative features are crucial for image classification and have a direct impact on performance, our approach to incorporate the spatial context by modeling relative relationship among triplets of identical visual words provides better results than the more recent feature-fusion based approaches.

The most significant results on UCM dataset, contributed by [46, 47], are 98.5% and 99.33% respectively. To the best of our knowledge, [47] provided the best classification for the UCM dataset. Prior to their work, the Penatti [57] achieved highest accuracy 93.4% with Caffenet, and 99.43% by combining Caffenet with OverFeat using SVM. Our proposed approach provides challenging results to the more recent highest performing deep neural networks based methods. A known tradeoff of deep CNN based architectures is that they typically contains millions of parameters for classification task and are difficult to train with limited training data. Despite of simple implementation, the proposed representation provides remarkable results for high resolution scene classification.

**Fig 15:**
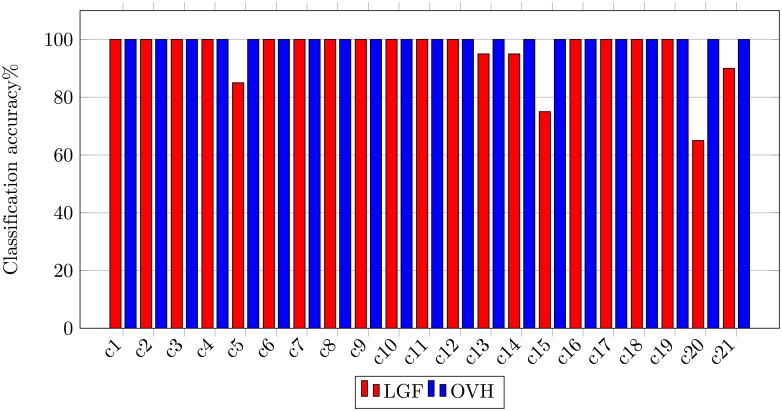
Class-wise comparison between LGF [1] and OVH for UCM image dataset.

In-order to demonstrate the sustainable performance of the proposed image representation, we have performed class-wise comparison with the state-of-the-art methods [1] shown in Fig 15. Experimental results using LGF show that the major confusion occurs between class overpass and intersection, and class storage and buildings. Our method successfully classifies all images to their respective categories thereby achieving 100% classification accuracy.

**Fig 16:**
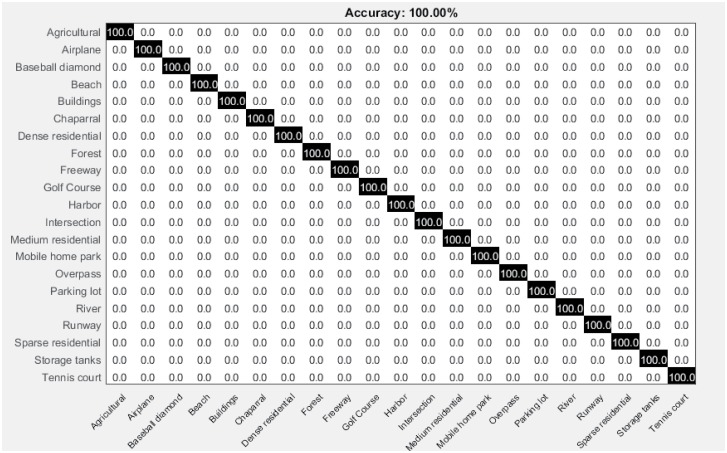
Confusion matrix for the UCM image dataset.

Fig 16 shows the average confusion matrix for UCM image dataset. It is clearly evident from the confusion matrix, that all the UCM classes are correctly classified achieving highest accuracy 100%.

### Performance on 19-class satellite scene image dataset

**Fig 17:**
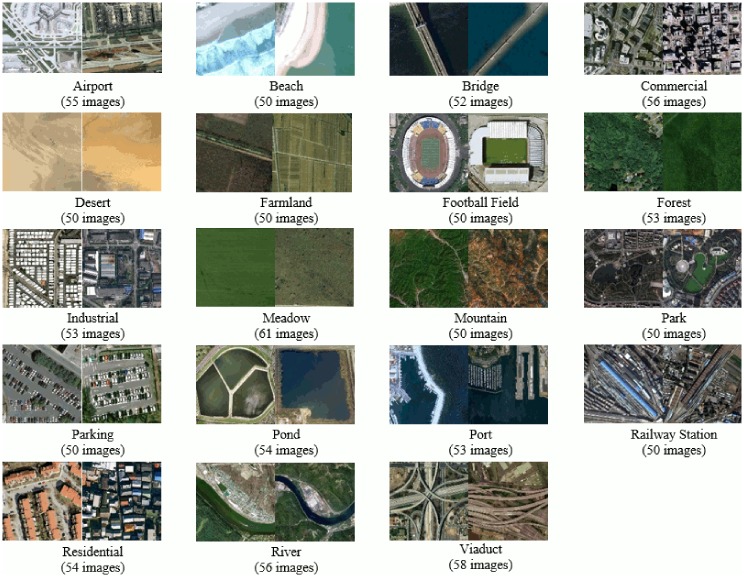
Example images with class label and total images per class, for each category of 19-class satellite scene image dataset [60].

The fourth dataset [1, 60] used in our experiments comprises of 19 high-resolution satellite scene categories as can be seen in Fig 17. This dataset focuses on images with a large geographical scale and contains atleast 50 images/class, size 600 × 600 pixels. Following the same experimental setup as in [1, 2], 30 images are chosen randomly from each class for training and the rest for testing.

**Fig 18:**
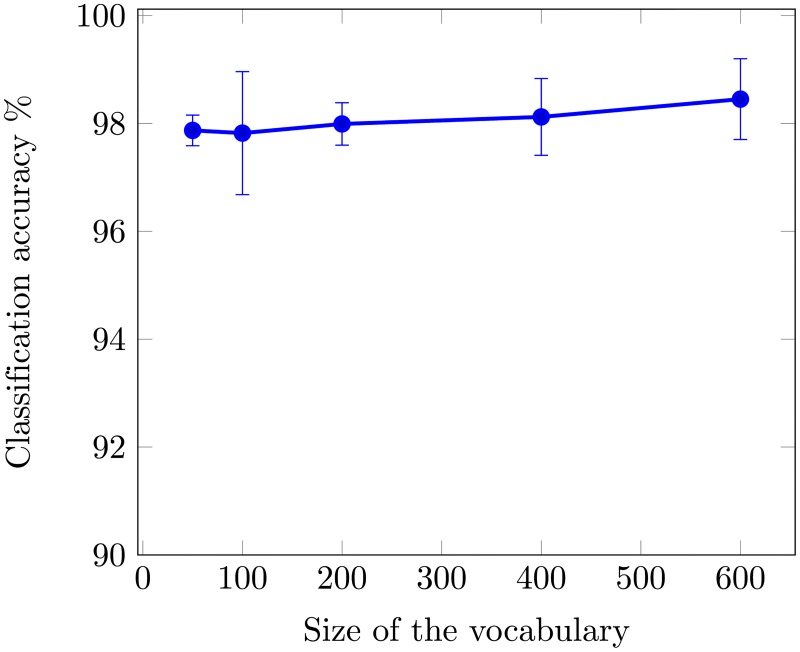
Mean average classification accuracy as a function of vocabulary size using 19-class satellite scene image dataset.

The performance of OVH, against different vocabulary sizes is shown in Fig 18. We obtained the optimal performance for a vocabulary of size 600, resulting in a 3000 dimensional histogram.

Table 5 provides a comparison of the proposed OVH to the state-of-the-art. It is important to mention here that we have not used BoVW, SPM [15] and related pioneer works in this sub-section for comparison, as our aim here is to provide a comparison with the recent outstanding reported works. The effectiveness of proposed approach to the concurrent methods has been shown in the above comparisons. It can be seen from Table 5 that our method shows competitive and reliable performance to the more recent state-of-the-art.

**Table 5:** Classification accuracy comparison of the proposed research with the state-of-the-art methods.

Algorithms	Accuracy
MS-CLBP_1_ [59]	93.4% ± 1.1%
LGF [1]	95.26%
salM^3^LBP-CLM [2]	96.38% ± 0.82%
GoogLeNet [46]	98.1%
OVH	98.45% ± 0.6%

**Fig 19:**
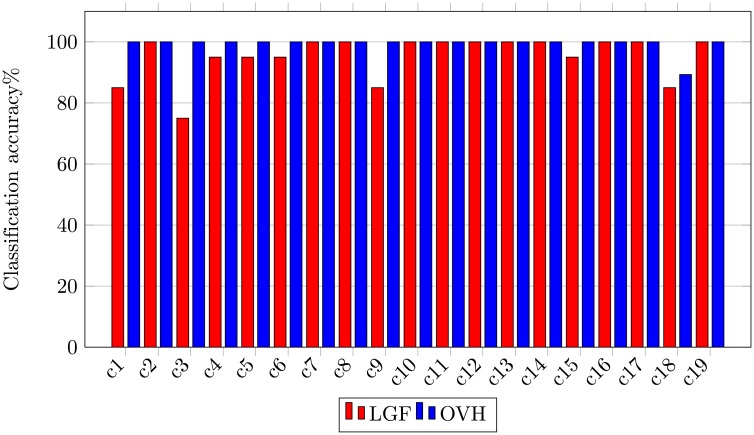
Class-wise comparison between LGF [1] and OVH for 19-class satellite scene image dataset.

MS-CLBP_1_ [59] is a multi-scale mutiresolution descriptor to capture dominant texture features applied for land-use scene classification. OVH provides 5.05% higher accuracy indicating its superiority for land-use scene classification. As mentioned earlier LGF [1] and salM^3^LBP-CLM [2] are local-global feature fusion methods. Compared to these method our approach provides 3.19% and 2.07% higher accuracy respectively. Our method provides 0.35% high accuracy compared to more recent deep network based GoogLeNet [46] method. The class-wise comparison between LGF [1] and OVH is shown in Fig 19, that allows the direct visualization of class-wise performance comparison between different methods.

**Fig 20:**
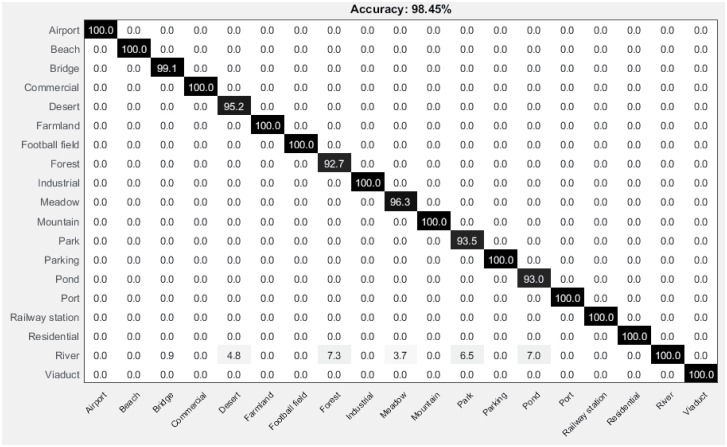
Confusion matrix for the 19-class satellite scene image dataset.

The confusion matrix for 19-class satellite scene image dataset is shown in Fig 20. Our proposed method to incorporate global spatial context, despite of its simple approach, shows remarkable performance compared to the state-of-the-art methods.

### Discussion on rotation-invariance

**Fig 21:**
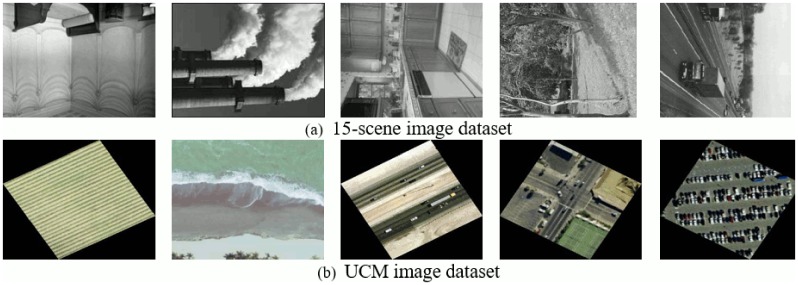
Example images from rotation datasets.

The OVH representation is invariant to rotation transformation. To demonstrate the effectiveness of proposed approach the analysis is performed on 15-scene and UCM image datasets. The selection of these datasets is made for two reasons. Firstly, of the four datasets used in our experiments these are larger in size. Secondly, the selected datasets have been in used literature to for rotation-invariance experiments, and hence a fair comparison is possible. Following the approach of Zhao *et al*. [31] and Karmakar *et al*. [61], a rotation dataset is created from the two datasets, by randomly rotating images. Example images from the rotation datasets are shown in Fig 21.

For 15-scene rotated image dataset the training and test ratio is in-consistent with one used for CCM-BOVW [31]. It is important note here that for classification accuracy comparison of UCM image dataset, the training and test images ratio is 0.8: 0.2 as in related works [1, 2]. Whereas for rotation-invariance experiments with rotated UCM dataset, the training test ratio of Zhao *et al*. [31] is followed i.e. 0.5: 0.5.

The experiments for rotation-invariance are performed for the optimal vocabulary size obtained from the classification experiments i.e. for 15-scene dataset at 400 (Fig 8) and 50 for UCM (Fig 14). Using the proposed OVH the mean accuracy obtained for 15-scene dataset is 84.52% and the dimensions of resultant feature vector are 2000. Karmakar *et al*. [61] proposed rotation-invariant SPM for image classification, and reported mean accuracy 83.4% with a 4200 dimensional feature vector. Our method provides 1.12% higher accuracy with dimensions less than half as compared to their work [61]. For our experiments we have used dense SIFT for feature extraction. It would be interesting to enhance the OVH feature representation by using a fusion of different techniques particulary with descriptors that could capture some rotation-invariance cues.

For the second rotated dataset, Zhao *et al*. [31] reported classification accuracy 86.64%, which exceeds the best accuracy reported by the dataset creator [56] by 5.45%. Our proposed method results in 100% classification accuracy with a 250 dimensional feature vector. OVH provides 13.36% higher accuracy compared to CCM-BOVW [31] method, which indicates that the proposed representation is very suitable to solve the land-use scene classification problem. CCM-BOVW didnot have a significant impact on the performance of classes that are relatively simple and do not suffer from orientations. Our method is equally beneficial for simple classes and also successfully classifies complex classes that are easily influenced by orientation e.g. storage tanks, baseball diamond, airplane, and golf course. To sum up, the proposed image representation is proved to be insensitive to the rotation of scenes.

## 5 Conclusion and future directions

In this paper, we proposed a novel low-dimensional image representation that incorporates the spatial information to the inverted index of BoVW model. The spatial information is added by calculating the global relative spatial orientation of visual words in a rotation invariant manner. This calculation provides the unique information regarding the relative position of visual words when they are collinear. We validated the proposed image representation by using four standard image benchmarks. The experimental results and quantitative comparisons demonstrate that our approach successfully incorporates relative global spatial information into the BoVW model. The proposed approach outperforms all other concurrent local and global histogram based methods and provides competitive performance as compared with more recent state-of-the-art approaches.

In future, we would like to extend this work to incorporate absolute spatial information, as the current trend shows combining these two in final representation is significant. For this we will enrich our representation by combining it with SPM or triangular histograms. As our method has shown excellent results on four image benchmarks, in future we would explore more challenging and large-scale datasets. Moreover, we intend to explore some new fuzzy encoding techniques with our triplet spatial histograms. To enrich our image representation with other cues like color and shape is also a promising direction for future research.
